# Size-controlled human adipose-derived stem cell spheroids hybridized with single-segmented nanofibers and their effect on viability and stem cell differentiation

**DOI:** 10.1186/s40824-021-00215-9

**Published:** 2021-04-26

**Authors:** Jinkyu Lee, Sangmin Lee, Sung Min Kim, Heungsoo Shin

**Affiliations:** 1 Department of Bioengineering, Hanyang University, Seoul, 04763 Republic of Korea; 2 BK21 FOUR, Human-Tech Convergence Program, Hanyang University, Seoul, 04763 Republic of Korea; 3 BK21 FOUR, Education and Research Group for Biopharmaceutical Innovation Leader, Hanyang University, Seoul, 04763 Republic of Korea; 4 Department of Physical Education and Active Aging Industry, Hanyang University, Seoul, 04763 Republic of Korea; 5 Center for Artificial Intelligence Muscle, Hanyang University, Seoul, 04763 Republic of Korea; 6 Institute of Nano Science and Technology, Hanyang University, Seoul, 04763 Republic of Korea

**Keywords:** Single segmented fibers, Spheroid, Stem cell, Angiogenic factor, Differentiation

## Abstract

**Background:**

Fabrication of three-dimensional stem cell spheroids have been studied to improve stem cell function, but the hypoxic core and limited penetration of nutrients and signaling cues to the interior of the spheroid were challenges. The incorporation of polymers such as silica and gelatin in spheroids resulted in relatively relaxed assembly of composite spheroids, and enhancing transport of nutrient and biological gas. However, because of the low surface area between cells and since the polymers were heterogeneously distributed throughout the spheroid, these polymers cannot increase the cell to extracellular matrix interactions needed to support differentiation.

**Methods:**

We developed the stem cell spheroids that incorporate poly(ι-lactic acid) single-segmented fibers synthesized by electrospinning and physical and chemical fragmentation. The proper mixing ratio was 2000 cells/μg fibers (average length of the fibers was 50 μm - 100 μm). The SFs were coated with polydopamine to increase cell binding affinity and to synthesize various-sized spheroids. The function of spheroids was investigated by in vitro analysis depending on their sizes. For statistical analysis, Graphpad Prism 5 software (San Diego, CA, USA) was used to perform one-way analysis of variance ANOVA with Tukey’s honest significant difference test and a Student’s *t*-test (for two variables) (*P* < 0.05).

**Results:**

Spheroids of different sizes were created by modulating the amount of cells and fibers (0.063 mm^2^–0.322 mm^2^). The fibers in the spheroid were homogenously distributed and increased cell viability, while cell-only spheroids showed a loss of DNA contents, internal degradation, and many apoptotic signals. Furthermore, we investigated stemness and various functions of various-sized fiber-incorporated spheroids. In conclusion, the spheroid with the largest size showed the greatest release of angiogenic factors (released VEGF: 0.111 ± 0.004 pg/ng DNA), while the smallest size showed greater effects of osteogenic differentiation (mineralized calcium: 18.099 ± 0.271 ng/ng DNA).

**Conclusion:**

The spheroids incorporating polydopamine coated single-segmented fibers showed enhanced viability regardless of sizes and increased their functionality by regulating the size of spheroids which may be used for various tissue reconstruction and therapeutic applications.

**Supplementary Information:**

The online version contains supplementary material available at 10.1186/s40824-021-00215-9.

## Background

Stem cells have been evaluated in tissue engineering due to their multipotent ability to differentiate into diverse tissue lineages, for example osteoblasts, neuronal cells, hepatocytes, and adipocytes [1, 2]. Adult stem cells (ASCs) can be isolated from various organs and tissues including bone marrow, turbinate, periosteum, and synovial membranes, and have been widely utilized for cell-based therapeutic approaches [3–5]. In particular, human adipose-derived stem cells (hADSCs) are an attractive source due to their ability to secrete diverse paracrine factors and their rapid doubling time of less than 70 h [6–8]. hADSCs have been used with direct injection [9, 10], in combination with scaffolds [11, 12] and as cell sheets [13, 14] to target tissues, such as skin and muscles. However, directly delivering stem cells without any scaffolds or cell related interactions are easily cleared or trapped in capillaries, limiting their therapeutic efficacy [15, 16]. Also, use of scaffolds can often activate potential inflammation and may reduce cell-cell interactions, leading to limited stemness [17–19]. Furthermore, mono-layered cell sheets have shown to be difficult to handle because of weak mechanical properties, and limit control of spontaneous and direct stem cell differentiation [20–22]. In addition, thick, multi-layered cell sheets limit oxygen and nutrient diffusion and the stem cells are unable to survive in vivo without blood vessels [23].

Alternatively, fabrication methods for three-dimensional (3D) cell spheroids have been studied as 3D micro-environment for cells. For instance, methods such as hanging drop, round micro-well, centrifugation, and a spinner flask with a bioreactor were mainly used for preparing spheroids [24–27]. Although these spheroids had improved stem cell function, there are still challenges in tissue engineering due to the hypoxic core and limited penetration of nutrients and signaling cues to the interior of the spheroid. By the reason, most of the spheroid diameters were less than 200 μm because larger-sized spheroids showed severe hypoxia from diffusion limitations [26, 28]. Severe hypoxia induces apoptosis, and it may limit the spheroid viability and survival time. So it was hard to expect the long survival for differentiation such as osteogenic or chondrogenic lineage which were needing at least 14 or 21 days of culturing under differentiation medium. The dynamic cultures in bioreactors could attenuate the hypoxia in spheroids and improve their viability, however, the fabricated spheroids showed heterogeneous sizes [29]. Also, spheroids composed only of cells could maintain their stemness because of high cell-cell interactions, but less cell- extracellular matrix (ECM) interactions may limit some differentiation, especially osteogenic differentiation in which cell-ECM interactions are an important factor [30].

The incorporation of polymers such as silica and gelatin in spheroids resulted in relatively the larger and relaxed assembly of composite spheroids because the polymer improved the viability of the spheroids by enhancing transport of nutrient and biological gas. However, because of the low surface area between cells and since the polymers were heterogeneously distributed throughout the spheroid, these polymers cannot increase the cell-ECM interactions needed to support differentiation [31–33]. Our previous study dealt with these issues by using homogeneously distributed fragmented nanofibers in spheroids and was successful in preventing hypoxia-related cell death and increased cellular function [26]. As the volume of spheroids increased, hypoxia was increased due to oxygen diffusion limitations and result in reducing osteogenic differentiation while increasing the release of angiogenic factors. These phenomenon have been demonstrated only in 2D cultures and not with a 3D culture system [34, 35]. Previous spheroids that had standardized sizes have been investigated regardless of the relation between the size and hypoxia induced stem cell reaction. Therefore, spheroids were showed limited sizes and functionally limited to one therapeutic outcome or differentiation; the spheroids could not be used in multi-functional approaches [36–38]. To successfully mimic the 3D micro-environment of the body, we needed to understand the different biological reaction of cells on spheroid from the change of sizes causing different hypoxia condition and customize the spheroids for diverse therapeutic approaches.

In this study, we synthesized single fragmented fibers (SFs) from an electrospun nanofiber sheet, hypothesizing that this method could increase spheroid viability, and prevent severe hypoxia, apoptosis, and diffusion limitations. To prove our hypothesis, we (1) evaluated the differences in spheroid morphology and viability and (2) investigated the difference of growth factor secretion and differentiation comparing variably-sized spheroids.

## Materials and methods

### Materials

poly(ι-lactic acid) (PLLA) was purchased from Samyang (Seoul, Korea). Trifluoroethanol, dopamine hydrochloride, anti-mouse IgG biotin conjugate, anti-rabbit IgG biotin conjugate, alizarin red S, and p-nitrophenyl phosphate were purchased from Sigma (St. Louis, MO, USA). Isopropyl alcohol, and Tris-HCl were obtained from EMD Millipore (Billerica, MA, USA) and Alfa Aesar (Heysham, UK), respectively. Fetal bovine serum (FBS), penicillin/streptomycin, and phosphate-buffered saline (PBS) were purchased from Wisent (St. Bruno, QC, Canada). The Quant-iT Picogreen dsDNA Assay kit was purchased from Invitrogen (Carlsbad, CA, USA), and the QuantiChrom Calcium Assay kit was purchased from BioAssay Systems (Hayward, CA, USA). Hematoxylin and eosin was purchased from BBC Biochemical (Mount Vernon, MA, USA). The Live and dead assay kit and streptavidin-FITC were obtained from Molecular Probes (Eugene, OR, USA) and ebioscience (San Diego, CA, USA), respectively. VEGF ELISA development kits were purchased from PeproTech (Rocky Hill, NJ, USA). The primary antibodies OCT4, NANOG, and SOX2 were purchased from Abcam (Cambridge, UK). Hypoxia Probe LOX-1 was purchased from SCIVAX Corporation (Kanagawa, Japan).

### Preparation and characterization of single-segmented fibers (SFs)

Thin nanofiber sheets were prepared by electrospinning using 10 ml of 4% PLLA in dichloromethane (DCM) and trifluoroethanol (TFE) (8,2, v/v). The solution was injected into a rotating collector (200 rpm) through a 23-G blunt end needle under 11–12 kV at 5 ml/hr. The electrospun sheet was then dried at room temperature overnight. To prepare the SFs, the sheet was cut into 5 × 5 mm pieces, which were subsequently treated with 10% (v/v) ethylenediamine (EDA) solution in isopropyl alcohol (IPA) and mixed for 30 min at 200 rpm on a parallel rotator. Fibers were then collected by centrifugation at 3000 rpm for 5 min and washed three times in IPA, once in 70% ethanol (EtOH), and three times in distilled water. The washed SFs were freeze-dried for 24 h. Field-emission scanning electron microscopy (FE-SEM) (AURIGA, Carl Zeiss, Germany) was used to observe the morphology of the SFs. In order to improve cell adhesion, SFs were rinsed once in 70% EtOH, three times with DW, and then immersed in a dopamine hydrochloride solution (2 mg/ml, 10 mM Tris-HCl buffer, pH 8.5) under mild shaking at 50 rpm for 10 min. Then, polydopamine-coated SFs (P-SFs) were washed three times with DW at 70 rpm for 10 min and lyophilized. The surface atomic compositions of SFs and P-SFs were analyzed with X-ray photoelectron spectroscopy (XPS) (Theta Probe base system; Thermo Fisher Scientific, Waltham, MA, USA).

### Culture of human adipose-derived stem cells

The hADSCs were purchased from Invitrogen (StemPro; Carlsbad, CA, USA). The cells were cultured under standard culture conditions (5% CO_2_ and 37 °C) in minimum essential medium, MesenPRO RS™ basal medium (Carlsbad, CA, USA) with 2% growth supplements (Carlsbad, CA, USA), 1% penicillin/streptomycin (P/S), and 1% L-glutamine. The cells were incubated in a 175 T culture flask and the medium was refreshed every 3 days. For the study, hADSCs that had attached to the bottom of the culture dish were detached by using 4 ml of 0.05% trypsin-ethylenediaminetetraacetic acid (EDTA). Cells surviving after the fourth passage were used for all experiments.

### Preparation of spheroids with P-SFs

To prepare the spheroids, P-SFs were sterilized with 70% EtOH 2 times under UV and subsequently washed three times with PBS. The hADSCs were trypsinized and approximately 1 × 10^4^, 2 × 10^4^, and 4 × 10^4^ cells were mixed in PCR tubes with different amounts of P-SFs (5 μg (FS-1), 10 μg (FS-2), and 20 μg (FS-4)). The cell mixtures were then centrifuged at 1200 rpm for 5 min and incubated for 24 h. The same number of hADSCs were also processed to prepare spheroids without the addition of P-SFs: CS-1, CS-2, and CS-4 for the 1 × 10^4^, 2 × 10^4^, and 4 × 10^4^ hADSC samples, respectively. Each spheroid was then placed in a 48-well culture plate pre-coated with filtered 2% agarose gel dissolved in distilled water (DW).

### Viability of cells within the spheroids

Live and dead assay kit was used to investigate the viability of cells within the spheroids at days 1 and 7. A working solution containing calcein AM (11000) and ethidium homodimer (1500) in DPBS were added to the spheroids and incubated for 20 min. Next, images were captured via fluorescence microscopy (TE2000) (Tokyo, Japan). The sizes of spheroids were measured by PhotoShop CS6 software (Adobe) (San Jose, CA, USA) from the captured images. DNA assays was performed to assess cell proliferation in the spheroids. The spheroids were collected at each time point, lysed in 100 μL of radioimmune precipitation assay (RIPA) lysis buffer (150 mM NaCl, 1% Triton X-100, 1% sodium deoxycholate, 0.1% SDS, 150 mM Tris, pH 7.2), and detected with the Quant-iT Picogreen dsDNA Assay kit according to the manufacturer’s instructions. Fluorescent intensity was measured using a spectrometer (VARIOSKAN LUX, Thermo Fisher Scientific) (Vantaa, Finland) with excitation and emission wavelengths of 480 to 520 nm.

### Histological analysis of the spheroids

The microscale images of the spheroid surface and interior were visualized using the sectioned spheroids. Transmission electron microscopy (TEM; LIBRA 120, Carl Zeiss, Oberkochen, Germany) was performed to visualize cell-fiber interactions within the spheroid. Briefly, spheroids cultured for 2 and 7 days were fixed in modified Karnovky’s fixation solution (2% paraformaldehyde and 2% glutaraldehyde in 0.05 M sodium cacodylate buffer, pH 7.2) and 1% osmium tetroxide (in 0.05 M sodium cacodylate buffer, pH 7.2) for 2 h each. The fixed spheroids were then treated with 2% uranyl acetate for membrane staining. Serial dehydration of fixed spheroids was carried out by incubating in increasing concentrations of EtOH (30 to 100%) followed by a transition step consisting of a 15 min treatment with 100% propylene oxide at room temperature. Samples were then embedded in Spurr’s resin at 70 °C for 24 h. Polymerized blocks of Spurr’s resin were sectioned with an ultra-microtome (MT-X; RMC, Tucson, AZ, USA). The spheroids were analyzed using TEM.

### Diffusion of fluorescent dye into the spheroids

For staining of the spheroid nuclei, Hoechst pentahydrate (bis-benzimide) (Invitrogen, Oregon, USA) nuclear staining dye was diluted in PBS to a 1 to 200 ratio. The solution (100 ml) was used to treat each spheroid for 2.5 h. The stained spheroids were cross-sectioned and images of the stained area were captured by confocal microscope (TCS SP5, Leica) (Hessen, Germany). For quantification, images of the whole area and unstained area of sectioned spheroids were analyzed by Photoshop CS6 to calculate the ratio of stained area.

### Analysis of the stemness of the hADSCs within the spheroids

Immunofluorescence (IF) staining and quantitative polymerase chain reaction (qPCR) were performed to assess expression of stemness markers, *octamer-binding transcription factor 4 (OCT4)*, *sex determining region Y-box 2 (SOX2),* and *NANOG*. The spheroids were evaluated on day 7. For IF staining, cryo-sectioned samples were fixed in 4% paraformaldehyde for 15 min followed by a 1 h treatment with blocking buffer (5% FBS, 0.1% Tween-20 in PBS). Subsequently, samples were incubated at 37 °C with primary antibody (1:100) overnight and then incubated with anti-mouse IgG biotin conjugate (1:200) for 1 h. This was followed by treatment with FITC-conjugated streptavidin (1:200) for 1 h. IF stained samples were then mounted using DAPI-containing mounting media and observed under a fluorescence microscope. For gene expression analysis, mRNA was extracted using a QIAGEN RNA extraction kit (QIAGEN, Hilden, Germany) following manufacturer’s instructions. RNA concentrations were estimated by measuring the absorbance at 260 nm using a nano-spectrometer (Nanodrop 2000; Thermo Scientific, Wilmington, DE, USA). The cDNA was synthesized from 1 μg of RNA using a Maxime RT PreMix kit from Intron Biotechnology (Seoul, Korea) and was processed in a Bio-Rad Thermocycler (Bio-Rad Laboratories, Hercules, CA, USA). Before RT-PCR was performed, 20 μl of cDNA solution, which was diluted with Ambion® DEPC-treated water (Life Technologies, Carlsbad, CA, USA), was mixed with 10 μl of SYBR® Premix Ex Taq™ (2X) Tli RNaseH Plus (Takara, Kusatsu, Japan), 0.4 μl of 10 pmol primers, 0.4 μl of ROX reference dye (50X), and 6.8 μl of DEPC-treated water. The sample solutions were processed in an RT-PCR StepOnePlus™ instrument (Life Technologies, Carlsbad, CA, USA). The amplification reaction was performed with denaturation at 95 °C for 10 min, followed by 40 cycles of annealing at 95 °C for 15 s and extension at 60 °C for 1 min. A melting curve stage was performed from 60.0 °C to 95.0 °C in increments of 0.5 °C per 5 s. To investigate fold changes of the spheroids, the hADSCs were cultured on tissue culture plate as a monolayer in parallel and then used as a control group. All reactions were conducted in triplicate. Primer sequences are listed in supporting file.

### LOX-1 staining and VEGF secretion

For LOX-1 staining, the working reagent (2 μmol LOX-1/L in growth media) was prepared and the spheroids were treated for overnight under standard culture conditions. To examine VEGF secretion, spheroids was cultured for 1, 4, and 7 days. Supernatants from each time point were collected and stored at − 20 °C. After collection, an enzyme-linked immunosorbent assay (ELISA) was performed using a VEGF ELISA development kit (PeproTech, Rocky Hill, NJ, USA) according to manufacturer’s instructions. RT-PCR for apoptosis-related genes (*B-cell lymphoma 2 (BCL-2)* and *Bcl-2-associated X protein (BAX))* was performed following the same procedure as described previously. All primer sequences are listed in supporting file.

### Osteogenic differentiation

Spheroids were cultured under osteogenic media for 14 days to assess differentiation. For visualization of the deposited calcium minerals, the spheroids were fixed with 4% paraformaldehyde, treated with Alizarin Red S (Sigma-Aldrich) (2% in DW with pH 4.2), and the images were captured by the microscope. The calcium ions were extracted by incubating the spheroids for overnight in 0.6 N HCL, and then the calcium assay was then performed according to the QuantiChrom Calcium Assay kit’s instructions. Quantitative PCR analysis of the osteogenic gene markers; *runt-related transcription factor 2 (Runx2)*, *osterix (OSX)*, *osteocalcin (OCN),* and *osteopontin (OPN)* was performed as following the same PCR protocols used for investigation of the stemness markers, as described in the previous section. Primers are listed in Table 1. To investigate fold changes of the spheroids, the hADSC monolayer was used as a control group.

**Table 1:** Primer sequences

Primers	Sequences
*GAPDH*	Fw: 5′- GTC AGT GGT GGA CCT GAC CT − 3′ Rv: 5′- TGC TGT AGC CAA ATT CGT TG − 3′
*OCT-4*	Fw: 5′- GCA GCG ACT ATG CAC AAC GA − 3′ Rv: 5′- CCA GAG TGG TGA CGG AGA CA − 3′
*NANOG*	Fw: 5′- CTA AGA GGT GGC AGA AAA ACA − 3′ Rv: 5′- CTG GTG GTA GGA AGA GTA AAG G − 3′
*SOX-2*	Fw: 5′- AGT TGG ACA GGG AGA TGG C − 3′ Rv: 5′- AAC CTT CCT TGC TTC CAC G − 3′
*BCL*	Fw: 5′- TTT GCT TCA GGG TTT CAT CC − 3′ Rv: 5′- CAG TTG AAG TTG CCG TCA GA − 3′
*BAX*	Fw: 5′- GGA TTG TGG CCT TCT TTG AG − 3′ Rv: 5′- TAA AGC CAG CCT CCG TTA TC − 3’
*RUNX-2*	Fw: 5’- GCA GTT CCC AAG CAT TTC AT − 3′ Rv: 5′- CAC TCT GGC TTT GGG AAG AG − 3’
*OSX*	Fw: 5’- TAA TGG GCT CCT TTC ACC TG − 3′ Rv: 5′- CAC TGG GCA GAC AGT CAG AA − 3’
*OCN*	Fw: 5’- GTG CAG AGT CCA GCA AAG GT − 3′ Rv: 5′- TCA GCC AAC TCG TCA CAG TC − 3’
*OPN*	Fw: 5’- TGA AAC GAG TCA GCT GGA TG − 3′ Rv: 5′- TGA AAT TCA TGG CTG TGG AA − 3’

### Statistical analysis

Quantitative data are expressed as means ± standard deviation. Graphpad Prism 5 software (San Diego, CA, USA) was used to perform one-way analysis of variance (ANOVA) with Tukey’s honest significant difference (HSD) test and a Student’s *t*-test (for two variables). Statistical significance was denoted as *P* < 0.05. All quantitative values were calculated from at least three samples (*n* ≥ 3). Experiments were independently replicated at least three times. We found that the trend of all results was the same each time.

## Results

**Fig. 1:**
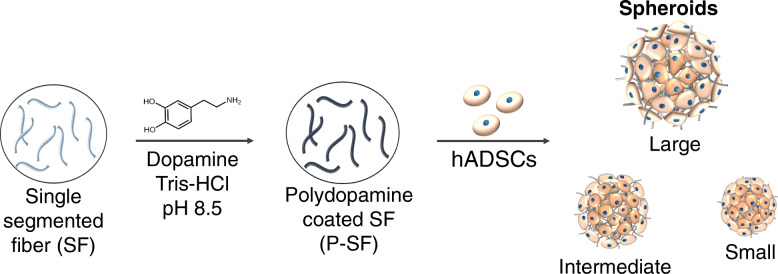
Schematic illustration of production methods for polydopamine-coated single-segmented fibers and various-sized spheroids

Multi-cellular spheroids from stem cells have been actively investigated in tissue engineering, however, the relatively hypoxic environment within the core may be detrimental to cell survival, which often limits their universal application. We prepared various sizes of hADSC spheroids by incorporating an artificial ECM from cell-adhesive single-segmented fibers (SF) and examined stem cell behavior within the spheroid. The SFs were coated with cell adhesive polydopamine (P-SFs) and co-assembled with hADSCs to form spheroids (Fig. 1). The objectives of this study were (1) to examine the effect of differing amounts of hADSCs and P-SFs on spheroid size, (2) to investigate the effect of P-SF on the viability of the spheroids, and (3) to investigate the effect of the spheroid size on secretion of pro-angiogenic factors and osteogenic differentiation.

### Preparation and characterization of the single-segmented fibers

**Fig. 2:**
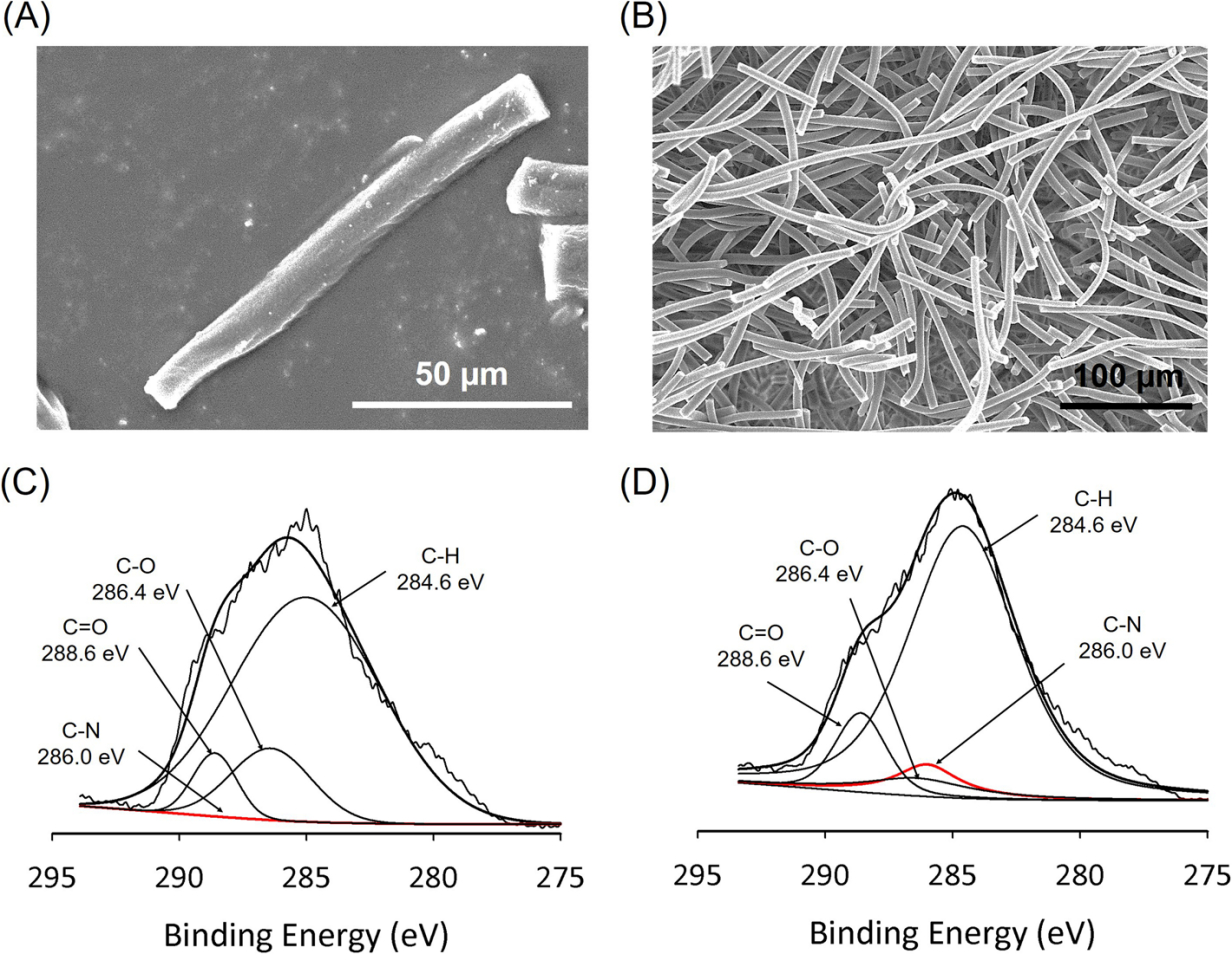
Characterization of the size and superficial chemical composition of SFs and P-SFs. SEM images of (**a**) single strand SFs and (**b**) aggregated SFs (scale bar: 50 and 100 μm). High-resolution XPS spectra of carbon peak C 1 s for (**c**) SF and (**d**) P-SF

SEM images showed the morphology of individual SFs (Fig. 2a) and the aggregated network (Fig. 2b). The length with 50 to 100 μm was selectively chosen by sieving and the average length of fiber was 68 ± 2 μm (Supplementary Figure 1). of the surface chemical composition using XPS revealed that the high-resolution carbon spectrum analysis revealed that the peak for the C-N bond (286.0 eV) on the surface of the fibers was 0.0096% in the SF group and 6.66% in the P-SF group (Fig. 2c and d), which indicated that the SFs were successfully coated with polydopamine.

### Preparation of size-controlled hADSCs spheroids

**Fig. 3:**
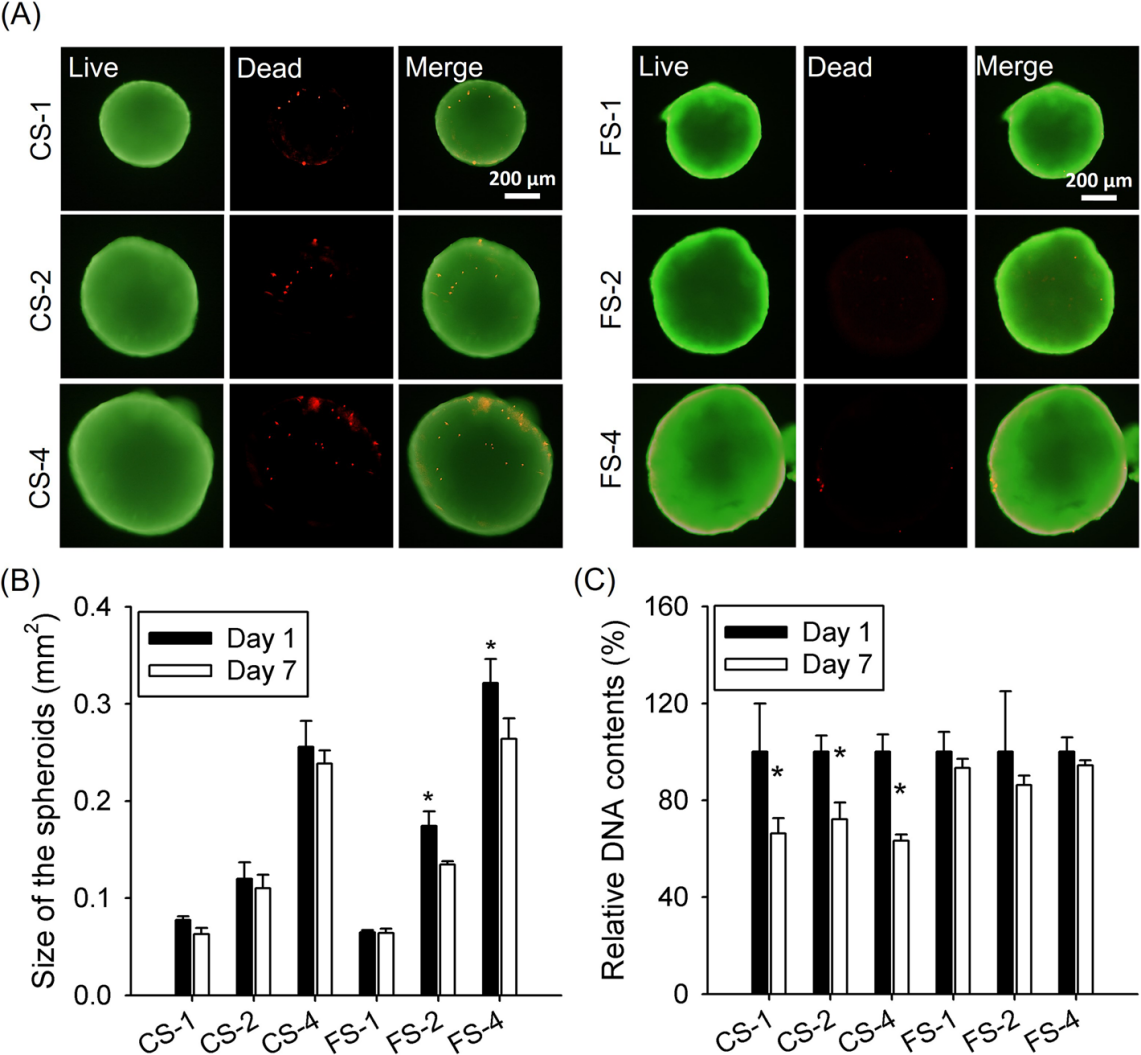
Fabrication of various-sized CS and FS groups. **a** Live and dead assay for both spheroid groups cultured for 7 days (scale bar: 200 μm). **b** The sizes of spheroids (“*” indicates a significant difference in sizes between 1 and 7 days), and (**c**) the DNA content of the spheroids on days 1 and 7 (“*” indicates a significant difference in DNA content between 1 and 7 days)

After synthesizing the P-SFs, we prepared the cell-only spheroids (CS-1, CS-2, and CS-4) and the fiber incorporated spheroids (FS-1, FS-2, and FS-4). The phase contrast images of the spheroids after 24 h from the centrifugation of cells and fibers showed that all spheroids were spherical shape and all the fibers and cells were well assembled to form the spheroid (Supplementary Figure 2). The live and dead assay at day 7 showed dead cell signals in the CS groups, particularly CS-4 while all FS groups showed comparatively minimal dead cells (Fig. 3a). The size of spheroids cultured for 24 h was well-controlled by the cell number within the spheroids. The area of spheroids prepared from 40,000 cells was the largest (0.256 ± 0.027 and 0.322 ± 0.025 mm^2^ for CS-4 and FS-4, respectively) while those of 10,000 cells showed the smallest area (0.078 ± 0.004 and 0.065 ± 0.002 mm^2^ for CS-1 and FS-1, respectively) (Fig. 3b). The DNA contents in the CS groups were decreased while the FS groups retained their DNA content during 7 days (Fig. 3c). DNA content on day 7 normalized to day 1 was 66.31 ± 6.43% (CS-1), 72.12 ± 7.05% (CS-2), 63.28 ± 2.52% (CS-4), 93.30 ± 3.80% (FS-1), 86.16 ± 3.94% (FS-2), and 94.38 ± 2.00% (FS-4).

### Histological analysis and diffusion of fluorescent dye

**Fig. 4:**
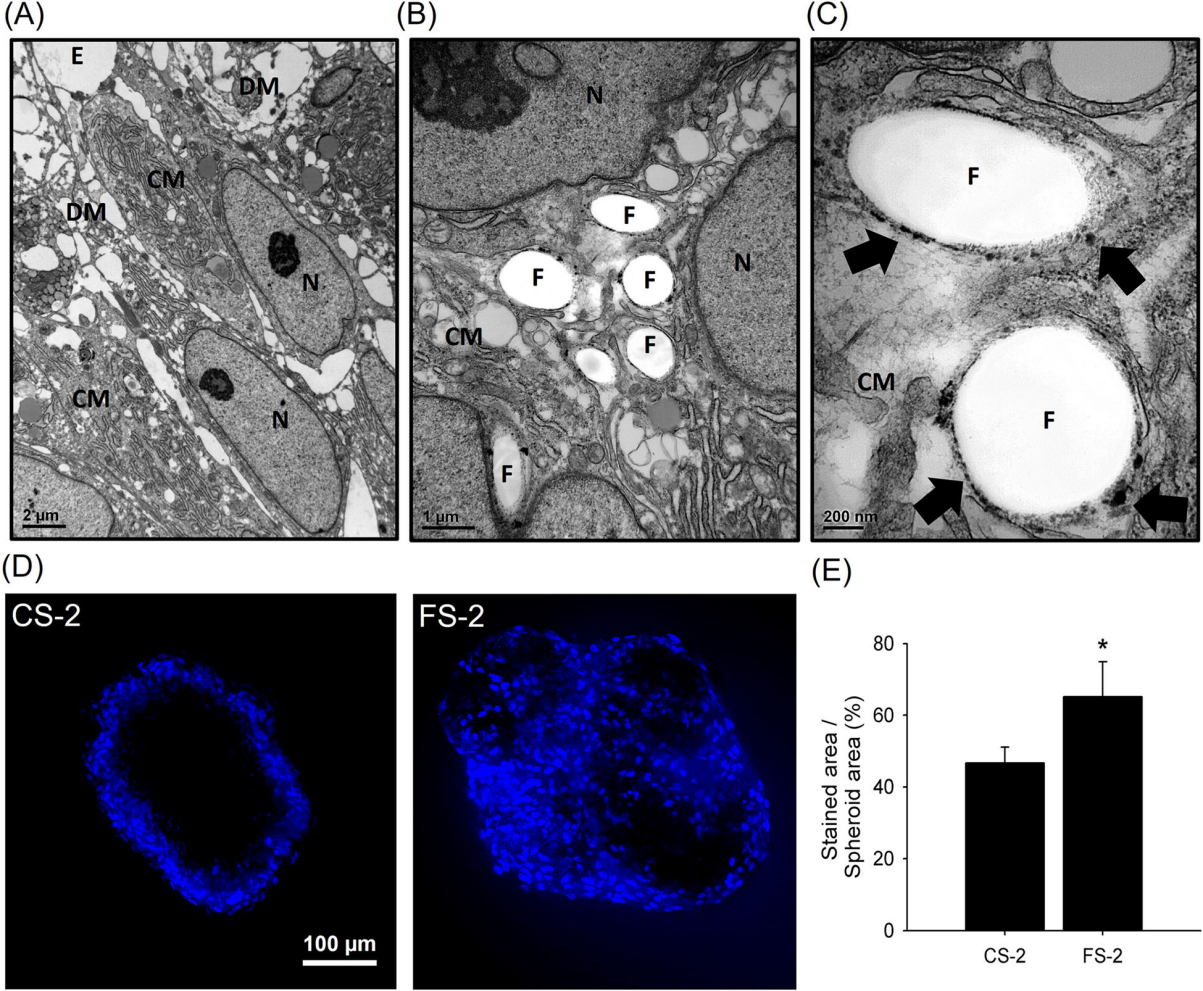
Histological analysis of spheroids and diffusion of fluorescent dye into spheroids. TEM microscopic images of cross-sectioned (**a**) CS-2 and (**b**) FS-2 groups, and (**c**) a high magnification image of the FS group (scale bar: 1 μm and 200 nm, respectively). (N: nucleus; E: empty space; CM: cell membrane; DM: disconnected membrane; F: single segmented fiber; black arrow: polydopamine particle). **d** Confocal microscope images after Hoechst staining of the CS and FS groups (scale bar: 100 μm). **e** Quantification of the percentage of stained areas

TEM images showed that CS-2 exhibited disintegrated membranes (DM) and empty spaces (E) (Fig. 4a). In contrast, the cell membranes (CM) in FS-2 were tightly bound with each other or with P-SFs (F), and disconnected or empty regions were minimal (Fig. 4b). The high magnification image clearly shows polydopamine particles (black arrows) on the surface of P-SFs and the fibers bound with adjacent cell membrane (Fig. 4c). To quantitate the difference in small molecule diffusion into the spheroid center (Fig. 4d), CS-2 and FS-2 were treated with Hoechst nuclear staining dye. The dye infiltration was much higher in FS-2, in which 65.13 ± 9.86% of the area was stained. The CS group showed only 46.63 ± 4.40% staining (Fig. 4e).

### Spheroid size and stemness

**Fig. 5:**
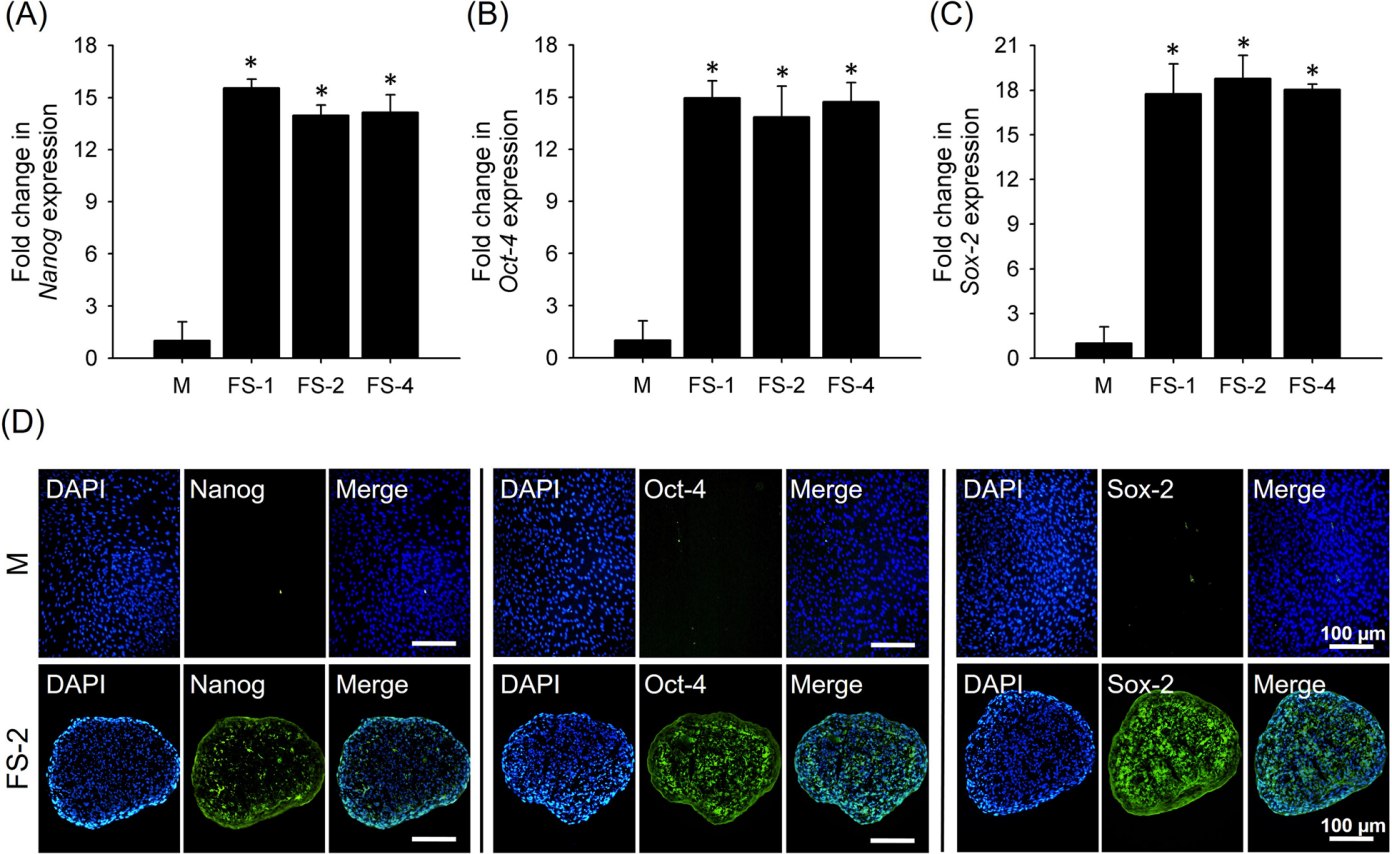
The stemness of FS-1, FS-2, and FS-4 groups. PCR analysis of the expression of (**a**) Nanog, (**b**) OCT4, and (**c**) SOX2 as compared to two dimensionally seeded cells (M) on day 7. **d** Immunostaining of all stemness markers from cross-sectioned FS-2 and group M spheroids (scale bar: 100 μm)

To investigate stemness maintenance of viable spheroids, all FS were cultured for 7 days, and the expression of the stemness markers *NANOG*, *OCT4,* and *SOX2* were evaluated. PCR analysis of the markers revealed significantly higher expression in the FS groups as compared to the cells which were seeded as mono-layered on tissue culture plate (M) group. FS-1 showed 15.54 ± 1.09, 14.96 ± 0.98, and 17.76 ± 2.01 fold increases for *NANOG*, *OCT4*, and *SOX2*, respectively (Fig. 5a, b and c). Despite the significant increase in stemness markers in spheroids over the group M, no significant difference in expression was found between the different spheroid sizes. In addition, high immunofluorescence signals for all markers were observed in FS-1, FS-2, and FS-4, while the group M showed less intense signal (Fig. 5d).

### Pro-angiogenic factor secretion of the spheroids

**Fig. 6:**
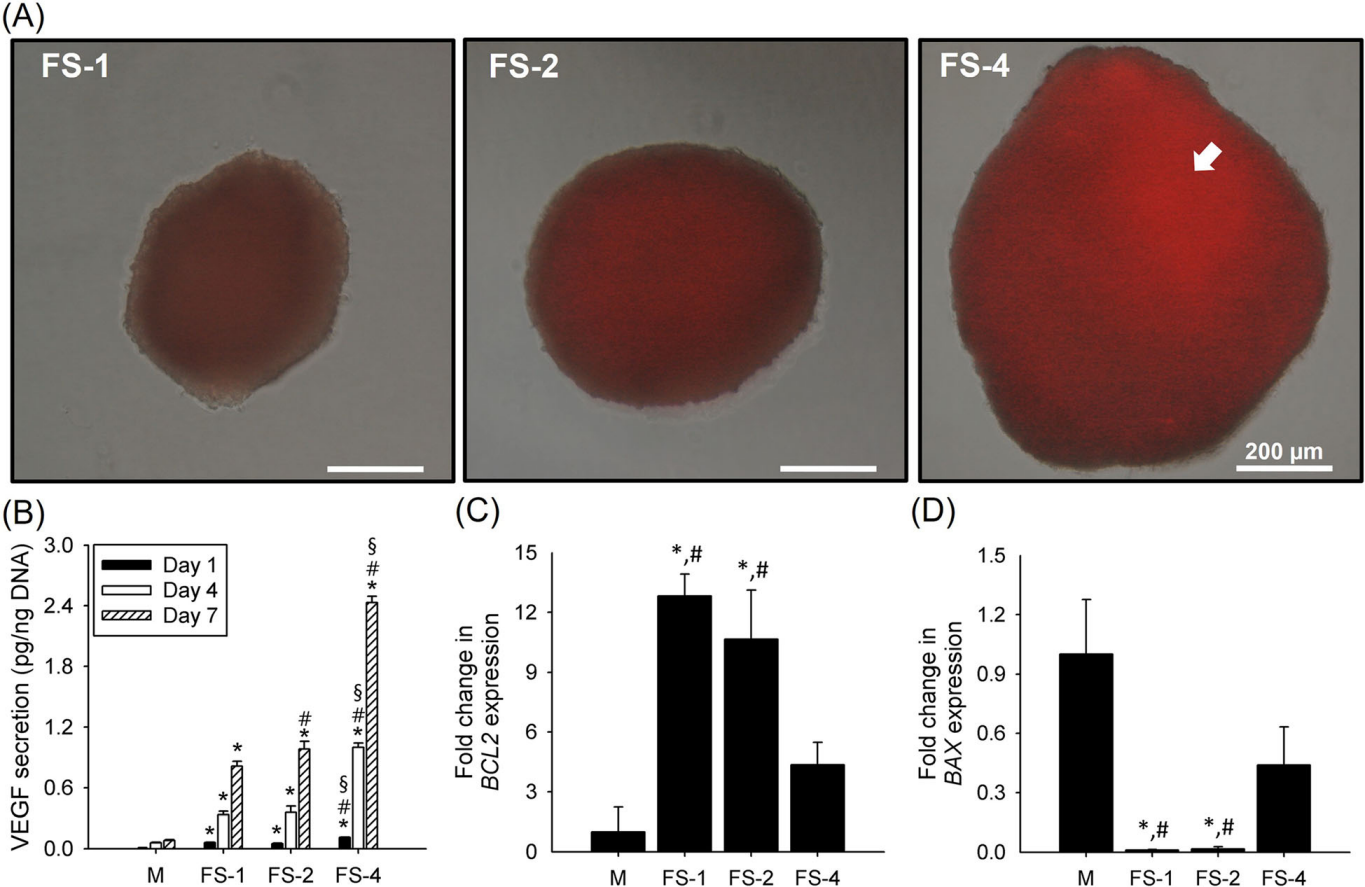
The secretion of angiogenic factor from the FS groups. **a** LOX-1 staining from FS-1, FS-2, and FS-4 (white arrow indicates a highly stained region in FS-4, scale bar: 200 μm). **b** The secreted amounts of VEGF from the all groups were quantified by ELISA (“*”, “#”, and “§” indicate significant differences between group M, FS-1, and FS-2, respectively, for each time point). PCR analysis of the expression of (**c**) BCL2 and (**d**) BAX genes in all groups

To examine the oxygen supply in the spheroids, the FS groups were cultured for 7 days and treated with LOX-1. FS-4 showed more intense red signal as compared to those from the other groups; the signal intensity decreased as spheroid size decreased (Fig. 6a). VEGF secretion had a similar trend to LOX-1 staining. VEGF secretion from FS-4 was 2.43 ± 0.06 pg/ng DNA on day 7, but it was significantly lower for the group M (0.08 ± 0.06 pg/ng DNA), FS-1 (0.81 ± 0.05 pg/ng DNA), and FS-2 (0.98 ± 0.08 pg/ng DNA) groups (Fig. 6b). We then analyzed the expression of the *BCL-2* and *BAX* genes. FS-1 showed the highest expression of *BCL-2* (12.8 ± 1.09 times greater than *BCL-2* expression in group M), and FS-4 showed the lowest expression of *BCL-2* (4.35 ± 1.13 times greater than *BCL-2* expression in group M). In contrast, *BAX* expression was reversed. With the exception of the control group M, FS-4 showed the highest *BAX* expression (0.44 ± 0.19 times lower than *BAX* expression in group M), and FS-1 showed the lowest *BAX* expression (0.010 ± 0.0029 times lower than *BAX* expression in group M) (Fig. 6c and d).

### Osteogenic differentiation of the spheroids

**Fig. 7:**
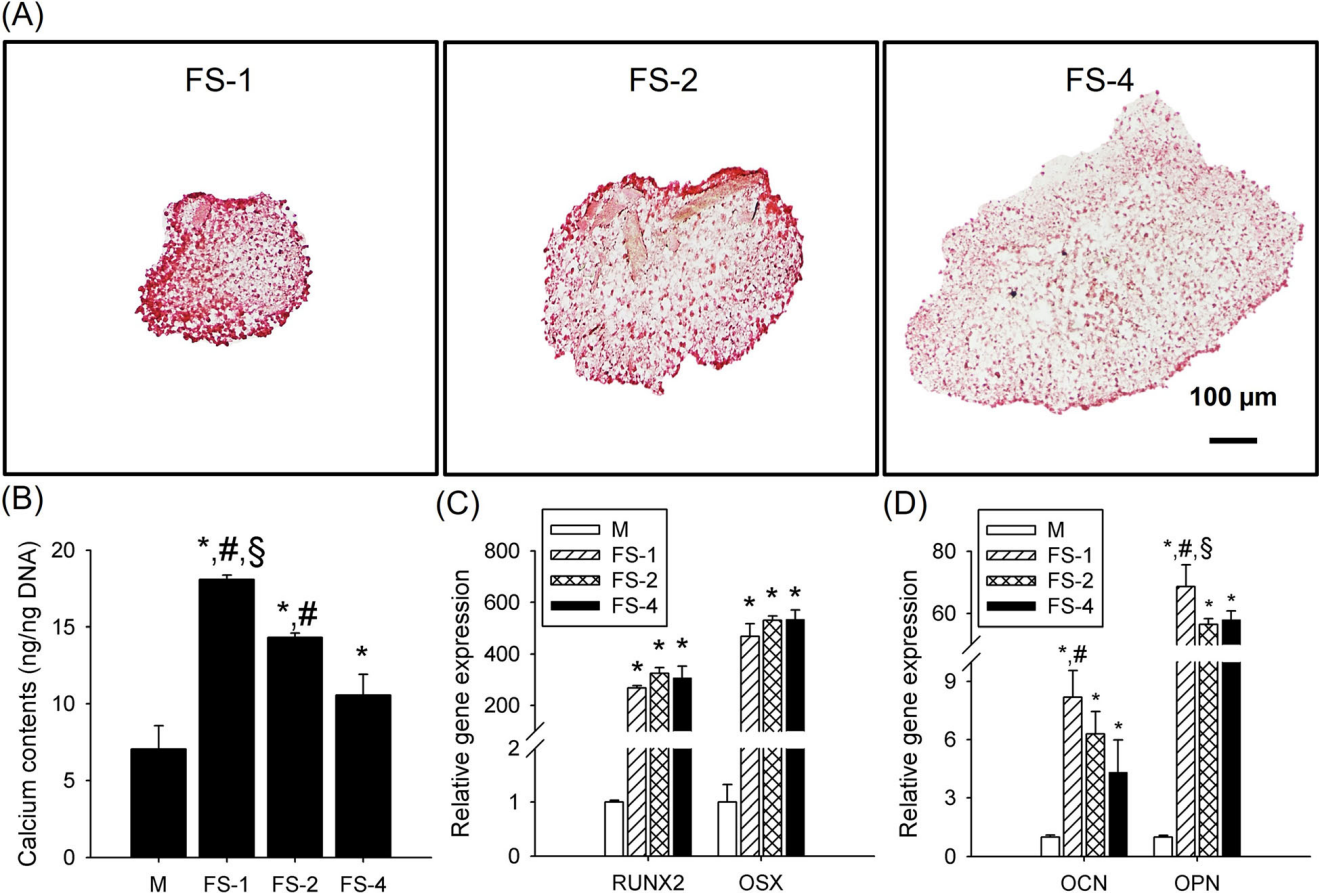
Osteogenic differentiation of the FS groups. **a** Alizarin red S staining (scale bar: 100 μm) and (**b**) a calcium assay (“*”, “#”, and “§” indicate significant differences between groups M, FS-4 and FS-2, respectively) were performed to investigate the amounts of mineralized calcium. **c** PCR analysis of osteogenic differentiation markers (**c**) RUNX2, OSX, (**d**) OCN, and OPN on day 7 (“*”, “#”, and “§” indicate significant differences between groups M, FS-2, and FS-4, respectively)

Finally, we investigated the osteogenic differentiation of FS via PCR analysis, a calcium assay, and staining. Alizarin red S staining resulted in mineralized calcium appearing as a reddish color and was more intense in FS-1 than in FS-4 (Fig. 7a). The quantification of mineralized calcium content revealed that the smaller spheroid contained significantly more calcium minerals (18.1 ± 0.27, 14.3 ± 0.28 and 10.6 ± 1.35 ng/ng DNA for FS-1, FS-2, and FS-4, respectively) (Fig. 7b). Furthermore, the expression of the initial and intermediate differentiation markers *RUNX2* and *OSX* were upregulated in the FS groups as compared to the control group (Fig. 7c). However, the expression of late differentiation markers such as *OCN* and *OPN* was the highest on FS-1 that the smallest one (*OCN* expression: 8.17 ± 1.38, 6.29 ± 1.16 and 4.31 ± 1.68 times higher than group M for FS-1, FS-2 and FS-4, respectively) (*OPN* expression: 68.62 ± 7.05, 56.44 ± 1.89 and 57.93 ± 2.81 times higher than group M for FS-1, FS-2 and FS-4, respectively) (Fig. 7d).

## Discussion

The aims of this study were to prepare various sizes of hADSC spheroids with ECM-mimicking SFs and to investigate the functional differences of stem cells. To prepare the spheroids, segmented fibers were selected ranging from 50 to 100 μm in length. When the fiber length was too long, the spheroid was not compact and loosely assembled, which resulted in inefficient spherical shape formation. As shown in Fig. 4b and c, P-SFs were homogenously distributed with cells and tightly incorporated with cells. Furthermore, FS could maintain its initial sphere shape through day 7, while previously studied polymer-containing spheroids [39, 40] could not maintain their initial shapes. These observations demonstrate that the P-SFs were appropriate for maintaining the overall spheroid shape and cell binding, akin to the role of the ECM in natural tissues [41, 42].

To investigate various cellular functions, we used hADSCs, which have the potential to differentiate into various cell types including osteocytes [43], adipocytes [44], chondrocytes [45], and neuronal cells [46]. In addition, hADSCs have a quicker doubling time than other adult stem cells. To stably assemble hADSCs with the fibers, the SFs were coated with polydopamine in order to enhance cell binding affinity. Previous reports have demonstrated that dopamine spontaneously polymerizes in a weak basic solution on the surface of diverse scaffolds via hydrophobic interaction and π-π stacking. This polydopamine layer allowed cell adhesion and absorption of serum proteins on the coated surface [47, 48]. When fibers exhibited insufficient cell adhesion properties, the spheroids would easily dissociate or take on an irregular shape during long term culture. For example, one previous study incorporating poly(lactic-co-glycolic acid) (PLGA) fragments with human embryonic kidney 293 cells which were cultured for 24 h showed many unmixed fibers outside of the spheroids [49]. The weak interactions between the fibers and cells within the spheroid may limit cell functions, for example mineralization, myofibroblast differentiation, cell migration, and proliferation [50, 51]. The P-SFs succeeded in binding with cells and providing space for oxygen and nutrient diffusion into the spheroids.

As demonstrated from Live/Dead and DNA assays (Fig. 3a and c), the CS groups showed several dead cell signals, and their DNA content was decreased after 7 days, while the FS groups successfully maintained the stem cell viability. A previous report had demonstrated that the internal cell death from apoptosis resulted in disconnected vacant space, and thus, spheroid size decreased and its morphology was changed [52], which was also confirmed on the TEM image of CS (Fig. 4a). Furthermore, the CS surface was arranged more compactly than that of FS (Fig. 4d), and this compact arrangement may limit penetration of biological gases and nutrients, leading to cell death [53]. Awadhesh N. Jha et al. had previously evaluated oxygen distribution within the cell spheroid, suggesting that there are discrete zones composed of outside, middle, and inside spheroid regions along the oxygen supply from high to low, exhibiting proliferating, quiescent viable and apoptotic core property, respectively. When a spheroid size is increased, the hypoxic zone would increase dramatically. For example, the spheroid with 350 ± 117 μm radius showed a 273 μm hypoxic zone and only a 77 μm viable zone [54]. Similarly, previous studies have reported that hypoxic conditions could cause loose of cell-cell binding, and the cell membrane degradation provides evidence supporting apoptosis [55–58]. Collectively, our results suggested that the presence of P-SFs within the spheroid improved the stem cell viability by mitigating the diffusion limitation of crucial survival factors including oxygen.

To be useful for stem cells, stemness should be well-maintained during the expansion period, and differentiation should be precisely controlled upon application [59–61]. As shown in Fig. 5, the 3D cultured FS spheroids showed the higher expression of stemness marker than the 2D cultured stem cells. This difference may be attributed to the complex microenvironment of cell aggregates recreated by the 3D cultured spheroid, which enhanced cell-to-cell interactions and induced cytoskeletal cellular reorganization, maintaining their multi-potent stemness [37]. Similar results were prevalent for other types of stem cells. For instance, spheroids composed not only with mesenchymal stem cells (MSCs) increased gene expression of diverse differentiation markers (*angiopoietin*, *bone morphogenetic proteins,* and *transforming growth factor beta-3*), but also spheroids composed of human umbilical cord stem cells showed up-regulation of stem cell markers, and adipogenic or osteogenic differentiation makers than those of 2D cultured cells [37, 62]. Similarly, our results demonstrated that FS-1, FS-2, and FS-4 significantly preserved stemness as compared to 2D cultured cells over the 7 days, regardless of their sizes. All FS groups had the same ratio of hADSCs to the amount of P-SFs along with homogeneous assembly which caused similar levels of cellular interactions and that may have led to similar stemness gene expression.

The spheroid cores of larger size may be subjected to low oxygen supply due to diffusion limitation (Fig. 6a). Our results showed that spheroids released greater amounts of pro-angiogenic growth factors as size increased (Fig. 6b). *BAX* expression showed a similar trend. These are consistent with previous studies that adipose-, bone marrow-, and umbilical cord-derived stem cells show greater secretion of pro-angiogenic factors and apoptotic gene expression under hypoxic conditions [63, 64]. Additionally, all spheroids revealed greater osteogenic marker expression than was seen in 2D cultured hADSCs (Fig. 7c and d). These results may be related to differing levels of oxygen supply in 2D cultured cells and spheroids. According to previous studies, the presence of stem cells under low oxygen availability stimulated expression of diverse growth factors such as *VEGF* and *TGF-beta*, which could increase osteogenic differentiation [35]. The factors significantly enhanced osteogenic differentiation in the spheroid groups as compared to the 2D cultured cells. Among the spheroid groups, there were no differences in the initial and intermediate osteogenic differentiation markers *RUNX2* and *OSX,* regardless of spheroid size; whereas late osteogenic markers *OCN* and *OPN,* which are related to mineralization and mineralized calcium content, showed significantly greater expression in the smallest spheroid (FS-1). This phenomenon may be related to different hypoxia levels of the spheroids. Previous studies have demonstrated that more severe hypoxic condition (2% oxygen) increases the expression of insulin growth factor binding protein (IGBP) in ADSCs and blocks insulin growth factor-1 (IGF-1) binding to its receptor (IGFR) on the cell membrane. This IGF-1 and IGFR binding has been shown to have important role in stem cell mineralization [34, 65]. The core of large sized spheroids (> 350 μm of radius) underwent similar level of hypoxia because of limited oxygen supply [54] despite the presence of engineered fibers (Fig. 6a) comparing with smaller sized one, so the smallest spheroid (FS-1) which were under the lowest hypoxia could show the highest capacity of osteogenic differentiation specifically amount of mineralized calcium contents and expression of *OCN* and *OPN* which were related with mineralization signaling pathway because FS-1 was more favored the IGF-1 and IGFR binding than the larger spheroids.

The spheroids described here that incorporate P-SFs address limitations of previous spheroids, specifically limited oxygen diffusion, viability, and cell-to-ECM interactions. Due to this approach, the spheroids could be dramatically increased in size (up to a radius of 700 μm). The variety of the sizes make it possible to support different stem cell functions, such as angiogenic factor secretion and osteogenic differentiation. Taken together, these spheroids may have the potential to be used for customized treatments.

## Conclusion

In this study, we prepared stem cell spheroids of various sizes by incorporating P-SFs and studied stem cell function within those spheroids. We demonstrated that the surface of fiber successfully coated with polydopamine, and analysis of the viability and histological experiments showed that the incorporation of fibers in a spheroid significantly enhanced stem cell viability by partially solving the diffusion limitation (FS showed 65.13 ± 9.86% dye infiltration, while CS showed 46.63 ± 4.40%). Also, we synthesized three different-sized spheroids using differing amounts of P-SFs and cells. The spheroids demonstrated similar expression of stemness markers, however, their differentiation capacities varied due to differences in hypoxia levels. The smallest FS showed 18.10 ± 0.27 times higher calcium content than the control groups; and higher osteogenic differentiation marker expression than larger-sized spheroids while released the lower amount of angiogenic factor. In contrast, the largest FS showed the lowest osteogenic differentiation capacity, but released the greatest amounts of VEGF (2.43 ± 0.06 pg/ng DNA) as compared to the control group (0.08 ± 0.06 pg/ng DNA). Taken together, our results suggest that biocompatible P-SFs can modulate the size of spheroids, make long-term spheroid culture possible, and may be important for tissue regeneration and therapeutic applications.

## Supplementary Information

**Additional file 1: Supplementary figure 1.** The initially synthesized single-segmented nanofibers were sieved to selectively choose the fibers with 50 to 100 mm, and the length of selected fibers were measured (*n*=10 to each range). **Supplementary figure 2.** The phase contrast images of spheroids after 24 hr from the centrifugation of cells and fibers.

## Data Availability

The datasets used and/or analyzed during the current study are available from the corresponding author on reasonable request.

## Abbreviations

3D: Three-dimensional
2D: Two-dimensional
PLLA: Poly(ι-lactic acid)
ASC: Adult stem cells
ECM: Extracellular matrix
SFs: Single-segmented fibers
P-SFs: SFs coated with polydopamine
CS: Cell-only spheroid
FS: Spheroid incorporating P-SFs
hADSCs: human adipose-derived stem cells

## References

[1] Biehl JK, Russell B (2009). Introduction to stem cell therapy. J Cardiovasc Nurs.

[2] Mirzaei H, Sahebkar A, Sichani LS, Moridikia A, Nazari S, Sadri Nahand J, Salehi H, Stenvang J, Masoudifar A, Mirzaei HR (2017). Therapeutic application of multipotent stem cells. J Cell Physiol.

[3] Kwon JS, Kim SW, Kwon DY, Park SH, Son AR, Kim JH, Kim MS (2014). In vivo osteogenic differentiation of human turbinate mesenchymal stem cells in an injectable in situ-forming hydrogel. Biomaterials.

[4] Lambrechts T, Papantoniou I, Viazzi S, Bovy T, Schrooten J, Luyten FP, Aerts JM (2016). Evaluation of a monitored multiplate bioreactor for large-scale expansion of human periosteum derived stem cells for bone tissue engineering applications. Biochem Eng J.

[5] de Sousa EB, Casado PL, Neto VM, Duarte MEL, Aguiar DP (2014). Synovial fluid and synovial membrane mesenchymal stem cells: latest discoveries and therapeutic perspectives. Stem Cell Res Ther.

[6] Tsuji W, Rubin JP, Marra KG (2014). Adipose-derived stem cells: implications in tissue regeneration. World J Stem Cells.

[7] Harasymiak-Krzyzanowska I, Niedojadlo A, Karwat J, Kotula L, Gil-Kulik P, Sawiuk M, Kocki J (2013). Adipose tissue-derived stem cells show considerable promise for regenerative medicine applications. Cell Mol Biol Lett.

[8] Frese L, Dijkman PE, Hoerstrup SP (2016). Adipose tissue-derived stem cells in regenerative medicine. Transfus Med Hemother.

[9] Spasovski D, Spasovski V, Bascarevic Z, Stojiljkovic M, Vreca M, Andjelkovic M, et al. Intra-articular injection of autologous adipose derived Mesenchymal stem cells in treatment of knee osteoarthritis. J Gene Med. 2017;20(1). 10.1002/jgm.3002. 10.1002/jgm.3002 29243283

[10] Li G, Miao F, Zhu J, Chen Y (2017). Antiangiogenesis gene therapy for hepatocellular carcinoma via systemic injection of mesenchymal stem cells engineered to secrete soluble Flt1. Mol Med Rep.

[11] Bhowmick S, Scharnweber D, Koul V (2016). Co-cultivation of keratinocyte-human mesenchymal stem cell (hMSC) on sericin loaded electrospun nanofibrous composite scaffold (cationic gelatin/hyaluronan/chondroitin sulfate) stimulates epithelial differentiation in hMSCs: in vitro study. Biomaterials.

[12] Ramirez-Rodriguez GB, Montesi M, Panseri S, Sprio S, Tampieri A (2017). Sandri M: (*) biomineralized recombinant collagen-based scaffold mimicking native bone enhances Mesenchymal stem cell interaction and differentiation. Tissue Eng A.

[13] Zhou W, Han C, Song Y, Yan X, Li D, Chai Z, Feng Z, Dong Y, Li L, Xie X, Chen F, Zhao Y (2010). The performance of bone marrow mesenchymal stem cell--implant complexes prepared by cell sheet engineering techniques. Biomaterials.

[14] Sukho P, Cohen A, Hesselink JW, Kirpensteijn J, Verseijden F, Bastiaansen-Jenniskens YM. Adipose tissue-derived stem cell sheet application for tissue healing in vivo: a systematic review. Tissue Eng Part B Rev. 2017. 10.1089/ten.TEB.2017.0142. 10.1089/ten.TEB.2017.0142 28665192

[15] Zhang J, Huang XW, Wang HJ, Liu XY, Zhang T, Wang YC, Hu DH (2015). The challenges and promises of allogeneic mesenchymal stem cells for use as a cell-based therapy. Stem Cell Res Ther.

[16] Nystedt J, Anderson H, Tikkanen J, Pietila M, Hirvonen T, Takalo R, Heiskanen A, Satomaa T, Natunen S, Lehtonen S (2013). Cell surface structures influence lung clearance rate of systemically infused mesenchymal stromal cells. Stem Cells.

[17] Alaribe FN, Manoto SL, Motaung SCKM (2016). Scaffolds from biomaterials: advantages and limitations in bone and tissue engineering. Biologia.

[18] Zhang S, Liu P, Chen L, Wang Y, Wang Z, Zhang B (2015). The effects of spheroid formation of adipose-derived stem cells in a microgravity bioreactor on stemness properties and therapeutic potential. Biomaterials.

[19] Farahani E, Patra HK, Jangamreddy JR, Rashedi I, Kawalec M, Pariti RKR, Batakis P, Wiechec E (2014). Cell adhesion molecules and their relation to (cancer) cell stemness. Carcinogenesis.

[20] Prata FP, Cerqueira MT, Moreira-Silva J, Pirraco RP, Reis RL, Marques AP (2014). Cryopreservation of cell sheets of adipose stem cells: limitations and successes. Tissue Eng Part A.

[21] Owaki T, Shimizu T, Yamato M, Okano T (2014). Cell sheet engineering for regenerative medicine: current challenges and strategies. Biotechnol J.

[22] Rayatpisheh S, Heath DE, Shakouri A, Rujitanaroj PO, Chew SY, Chan-Park MB (2014). Combining cell sheet technology and electrospun scaffolding for engineered tubular, aligned, and contractile blood vessels. Biomaterials.

[23] Zhang LJ, Xing Q, Qian ZC, Tahtinen M, Zhang ZQ, Shearier E, Qi SH, Zhao F (2016). Hypoxia created human Mesenchymal stem cell sheet for Prevascularized 3D tissue construction. Adv Healthc Mat.

[24] Gong X, Lin C, Cheng J, Su JS, Zhao H, Liu TL, et al. Generation of multicellular tumor spheroids with microwell-based agarose scaffolds for drug testing. Plos One. 2015;10(6). 10.1371/journal.pone.0130348. 10.1371/journal.pone.0130348 PMC4474551 26090664

[25] Kuo CT, Wang JY, Lin YF, Wo AM, Chen BPC, Lee H. Three-dimensional spheroid culture targeting versatile tissue bioassays using a PDMS-based hanging drop array. Sci Rep. 2017;7(1). 10.1038/S41598-017-04718-1. 10.1038/s41598-017-04718-1 PMC5491519 28663555

[26] Ahmad T, Lee J, Shin YM, Shin HJ, Perikamana SKM, Park SH, Kim SW, Shin H (2017). Hybrid-spheroids incorporating ECM like engineered fragmented fibers potentiate stem cell function by improved cell/cell and cell/ECM interactions. Acta Biomater.

[27] Achilli TM, Meyer J, Morgan JR (2012). Advances in the formation, use and understanding of multi-cellular spheroids. Expert Opin Biol Ther.

[28] Laschke MW, Schank TE, Scheuer C, Kleer S, Shadmanov T, Eglin D, Alini M, Menger MD (2014). In vitro osteogenic differentiation of adipose-derived mesenchymal stem cell spheroids impairs their in vivo vascularization capacity inside implanted porous polyurethane scaffolds. Acta Biomater.

[29] Massai D, Isu G, Madeddu D, Cerino G, Falco A, Frati C, et al. A versatile bioreactor for dynamic suspension cell culture. application to the culture of cancer cell spheroids. Plos One. 2016;11(5). 10.1371/journal.pone.0154610. 10.1371/journal.pone.0154610 PMC4856383 27144306

[30] Baraniak PR, McDevitt TC (2012). Scaffold-free culture of mesenchymal stem cell spheroids in suspension preserves multilineage potential. Cell Tissue Res.

[31] Lou YR, Kanninen L, Kaehr B, Townson JL, Niklander J, Harjumaki R, et al. Silica bioreplication preserves three-dimensional spheroid structures of human pluripotent stem cells and HepG2 cells. Sci Rep. 2015;5(1). 10.1038/Srep13635. 10.1038/srep13635 PMC4555166 26323570

[32] Baraniak PR, Cooke MT, Saeed R, Kinney MA, Fridley KM, McDevitt TC (2012). Stiffening of human mesenchymal stem cell spheroid microenvironments induced by incorporation of gelatin microparticles. J Mech Behav Biomed Mater.

[33] Wei JJ, Lu JF, Liu YW, Yan SL, Li XH (2016). Spheroid culture of primary hepatocytes with short fibers as a predictable in vitro model for drug screening. J Mater Chem B.

[34] Qiu YY, Chen Y, Zeng TH, Guo WZ, Zhou WY, Yang XJ (2016). EGCG ameliorates the hypoxia-induced apoptosis and osteogenic differentiation reduction of mesenchymal stem cells via upregulating miR-210. Mol Biol Rep.

[35] Binder BYK, Sagun JE, Leach JK (2015). Reduced serum and hypoxic culture conditions enhance the Osteogenic potential of human Mesenchymal stem cells. Stem Cell Rev Rep.

[36] Murphy KC, Hoch AI, Harvestine JN, Zhou DJ, Leach JK (2016). Mesenchymal stem cell spheroids retain Osteogenic phenotype through alpha (2) beta (1) signaling. Stem Cells Transl Med.

[37] Zhang SC, Liu P, Chen L, Wang YJ, Wang ZG, Zhang B (2015). The effects of spheroid formation of adipose-derived stem cells in a microgravity bioreactor on sternness properties and therapeutic potential. Biomaterials.

[38] Heiss M, Hellstrom M, Kalen M, May T, Weber H, Hecker M, Augustin HG, Korff T (2015). Endothelial cell spheroids as a versatile tool to study angiogenesis in vitro. FASEB J.

[39] Kirn TG, Park SH, Chung HJ, Yang DY, Park TG (2010). Hierarchically assembled Mesenchymal stem cell spheroids using biomimicking Nanofilaments and microstructured scaffolds for vascularized adipose tissue engineering. Adv Funct Mater.

[40] Yamada M, Hori A, Sugaya S, Yajima Y, Utoh R, Yamato M, Seki M (2015). Cell-sized condensed collagen microparticles for preparing microengineered composite spheroids of primary hepatocytes. Lab Chip.

[41] Song M, Liu Y, Hui L (2018). Preparation and characterization of acellular adipose tissue matrix using a combination of physical and chemical treatments. Mol Med Rep.

[42] Nisbet DR, Forsythe JS, Shen W, Finkelstein DI, Horne MK (2009). Review paper: a review of the cellular response on electrospun Nanofibers for tissue engineering. J Biomater Appl.

[43] de Girolamo L, Sartori MF, Albisetti W, Brini AT (2007). Osteogenic differentiation of human adipose-derived stem cells: comparison of two different inductive media. J Tissue Eng Regen Med.

[44] Zou J, Wang WW, Neffe AT, Xu X, Li ZD, Deng ZJ, Sun XL, Ma N, Lendlein A (2017). Adipogenic differentiation of human adipose derived mesenchymal stem cells in 3D architectured gelatin based hydrogels (ArcGel). Clin Hemorheol Microcirc.

[45] Goh BS, Omar SNC, Ubaidah MA, Saim L, Sulaiman S, Chua KH (2017). Chondrogenesis of human adipose derived stem cells for future microtia repair using co-culture technique. Acta Otolaryngol.

[46] Gao S, Zhao P, Lin C, Sun YX, Wang YL, Zhou ZC, Yang DJ, Wang XL, Xu HZ, Zhou F, Cao L, Zhou W, Ning K, Chen X, Xu J (2014). Differentiation of human adipose-derived stem cells into neuron-like cells which are compatible with Photocurable three-dimensional scaffolds. Tissue Eng A.

[47] Perikamana SKM, Lee J, Lee YB, Shin YM, Lee EJ, Mikos AG, Shin H (2015). Materials from mussel-inspired chemistry for cell and tissue engineering applications. Biomacromolecules.

[48] Hiraishi N, Kaneko D, Taira S, Wang SQ, Otsuki M, Tagami J. Mussel-mimetic, bioadhesive polymers from plant-derived materials. J Invest Clin Dent. 2015;6(1). 10.1111/jicd.12054. 10.1111/jicd.12054 23857900

[49] Shin JY, Park J, Jang HK, Lee TJ, La WG, Bhang SH, Kwon IK, Kwon OH, Kim BS (2012). Efficient formation of cell spheroids using polymer nanofibers. Biotechnol Lett.

[50] Ma W, Tavakoli T, Derby E, Serebryakova Y, Rao MS, Mattson MP (2008). Cell-extracellular matrix interactions regulate neural differentiation of human embryonic stem cells. BMC Dev Biol.

[51] Naugle JE, Olson ER, Zhang XJ, Mase SE, Pilati CF, Maron MB, Folkesson HG, Horne WI, Doane KJ, Meszaros JG (2006). Type VI collagen induces cardiac myofibroblast differentiation: implications for postinfarction remodeling. Am J Phys Heart Circ Phys.

[52] Nunez R, Sancho-Martinez SM, Novoa JML, Lopez-Hernandez FJ (2010). Apoptotic volume decrease as a geometric determinant for cell dismantling into apoptotic bodies. Cell Death Differ.

[53] Cesarz Z, Tamama K (2016). Spheroid culture of Mesenchymal stem cells. Stem Cells Int.

[54] Langan LM, Dodd NJF, Owen SF, Purcell WM, Jackson SK, Jha AN. Direct measurements of oxygen gradients in spheroid culture system using electron parametric resonance oximetry. Plos One. 2016;11(2). 10.1371/journal.pone.0149492. 10.1371/journal.pone.0149492 PMC4764677 26900704

[55] Lai ZB, Kalkunte S, Sharma S (2011). A critical role of Interleukin-10 in modulating hypoxia-induced preeclampsia-like disease in mice. Hypertension.

[56] Vasilevskaya IA, Selvakumaran M, Roberts D, O'Dwyer PJ (2016). JNK1 inhibition attenuates hypoxia-induced autophagy and sensitizes to chemotherapy. Mol Cancer Res.

[57] Fink SL, Cookson BT (2005). Apoptosis, pyroptosis, and necrosis: mechanistic description of dead and dying eukaryotic cells. Infect Immun.

[58] Noto A, Raffa S, De Vitis C, Roscilli G, Malpicci D, Coluccia P, Di Napoli A, Ricci A, Giovagnoli MR, Aurisicchio L (2013). Stearoyl-CoA desaturase-1 is a key factor for lung cancer-initiating cells. Cell Death Dis.

[59] Kessler M, Hoffmann K, Brinkmann V, Thieck O, Jackisch S, Toelle B, Berger H, Mollenkopf HJ, Mangler M, Sehouli J, Fotopoulou C, Meyer TF (2015). The notch and Wnt pathways regulate stemness and differentiation in human fallopian tube organoids. Nat Commun.

[60] Jeong Y, Mangelsdorf DJ (2009). Nuclear receptor regulation of stemness and stem cell differentiation. Exp Mol Med.

[61] Sbrana FV, Cortini M, Avnet S, Perut F, Columbaro M, De Milito A, Baldini N (2016). The role of autophagy in the maintenance of Stemness and differentiation of Mesenchymal stem cells. Stem Cell Rev Rep.

[62] Li Y, Guo G, Li L, Chen F, Bao J, Shi YJ, Bu H (2015). Three-dimensional spheroid culture of human umbilical cord mesenchymal stem cells promotes cell yield and stemness maintenance. Cell Tissue Res.

[63] Nan L, Xu LA (2013). The hypoxia-induced secretion of PDGF-BB by Hepatocellular Carcinoma Cells Increases Activated Hepatic Stellate Cell Proliferation, Migration and VEGF-A Expression. Hepatology.

[64] Hu DN, Rosen RB, Iacob CE (2015). Hypoxia induces VEGF secretion in uveal melanocytes through increased protein levels of hypoxia-inducible factors-1 alpha. Invest Ophthalmol Visual Sci.

[65] Kim JH, Yoon SM, Song SU, Park SG, Kim WS, Park IG, et al. Hypoxia Suppresses Spontaneous Mineralization and Osteogenic Differentiation of Mesenchymal Stem Cells via IGFBP3 Up-Regulation. Int J Mol Sci. 2016;17(9). 10.3390/Ijms17091389. 10.3390/ijms17091389 PMC5037669 27563882

