# Supplementary material for: Size-controlled human adipose-derived stem cell spheroids hybridized with single-segmented nanofibers and their effect on viability and stem cell differentiation
Source: Biomater Res. 2021 Apr 26;25:14. doi: 10.1186/s40824-021-00215-9 (PMC8074457; doi:10.1186/s40824-021-00215-9)
Supplement: Supplementary file 1 — Additional file 1: Supplementary figure 1. The initially synthesized single-segmented nanofibers were sieved to selectively choose the fibers with 50 to 100 mm, and the length of selected fibers were measured (n=10 to each range). Supplementary figure 2. The phase contrast images of spheroids after 24 hr from the centrifugation of cells and fibers. [file 40824_2021_215_MOESM1_ESM.docx]

**Supplementary Data**

**Size-controlled human adipose-derived stem cell spheroids hybridized with single-segmented nanofibers and their effect on viability and stem cell differentiation**

Jinkyu Lee^1, 2^, Sangmin Lee^1, 3^, Sung Min Kim^2, 4, 5*^, and Heungsoo Shin^1, 3, 6*^

^1^Department of Bioengineering, Hanyang University, Seoul 04763, Republic of Korea

^2^BK21 FOUR, Human-Tech Convergence Program, Hanyang University, Seoul 04763, Republic of Korea

^3^BK21 FOUR, Education and Research Group for Biopharmaceutical Innovation Leader, Hanyang University, Seoul 04763, Republic of Korea

^4^Department of Physical Education and Active Aging Industry, Hanyang University, Seoul 04763, Republic of Korea

^5^Center for Artificial Intelligence Muscle, Hanyang University, Seoul 04763, Republic of Korea

^6^Institute of Nano Science and Technology, Hanyang University, Seoul 04763, Republic of Korea

*Co-corresponding author:

Sung Min Kim, Ph.D.

Tel: +82-2220-2880

E-mail: [minarthur@hanyang.ac.kr](mailto:minarthur@hanyang.ac.kr)

Heungsoo Shin, Ph.D.

Tel: +82-2-2220-2346

Fax: +82-2-2298-2346

E-mail: [hshin@hanyang.ac.kr](mailto:hshin@hanyang.ac.kr)


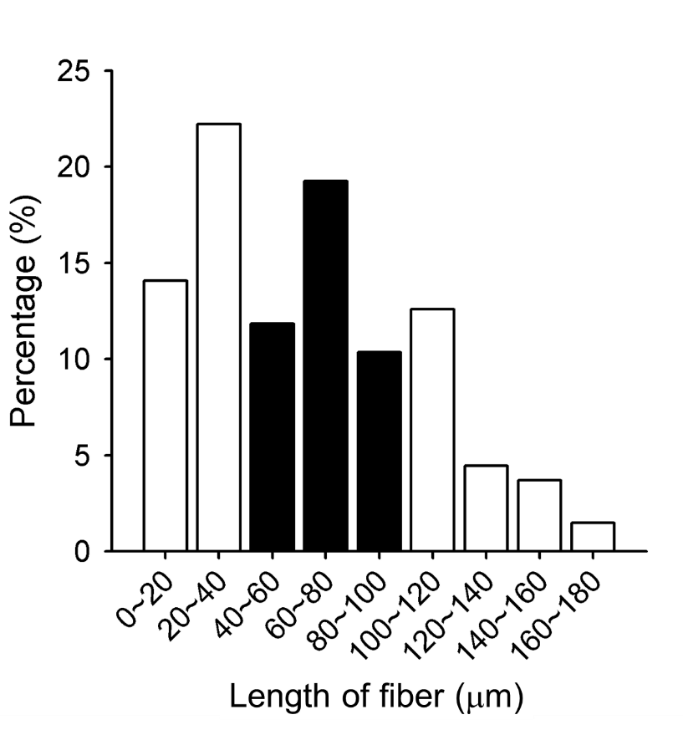


Supplementary figure 1. The initially synthesized single-segmented nanofibers were sieved to selectively choose the fibers with 50 to 100 mm, and the length of selected fibers were measured (n=10 to each range).


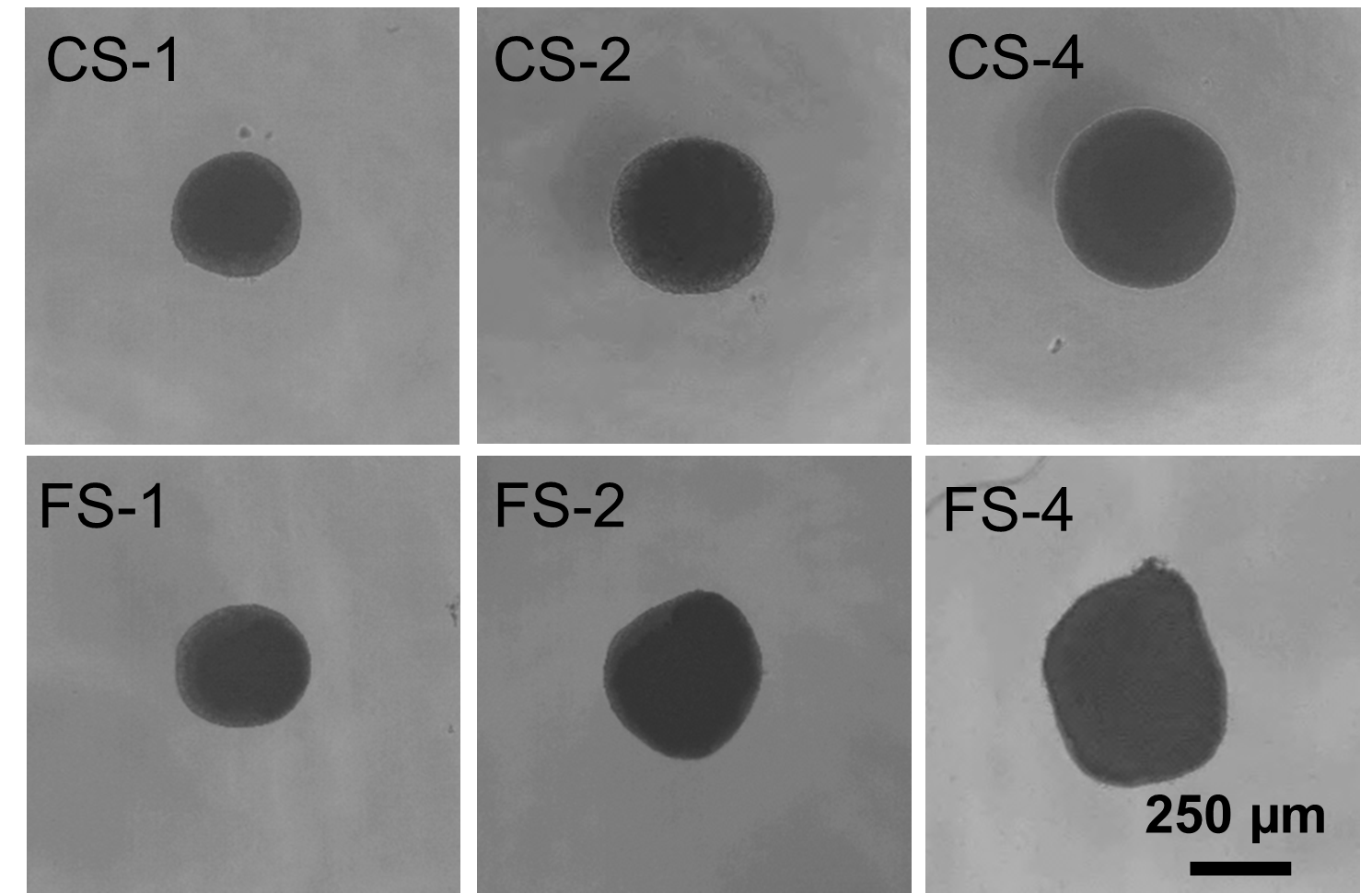


Supplementary figure 2. The phase contrast images of spheroids after 24 hr from the centrifugation of cells and fibers.
